# Significant changes in resuscitation guidelines: current and future recommendations?

**Published:** 2008-10

**Authors:** Walter GJ Kloeck

**Affiliations:** Resuscitation Council of Southern Africa, and College of Emergency Medicine of South Africa

Since the first description of cardiopulmonary resuscitation in 1960, a cumulative meta-analysis of published outcomes after pre-hospital cardiac arrest 40 years later showed that survival is still dismal, and is generally less than 6%.^1^ Furthermore, an analysis of mortality rates over a 19-year period has shown that survival rates have not been improving.^2^ Clearly, dramatic changes to recommendations on cardiovascular resuscitation need to be made.

In the most comprehensive review of resuscitation literature ever performed, involving more than 380 experts from 18 different countries, covering 276 different topics over a 36-month period, the International Liaison Committee on Resuscitation (ILCOR), represented by the American Heart Association, the Heart and Stroke Foundation of Canada, the Inter-American Heart Foundation, the European Resuscitation Council, the Australia and New Zealand Committee on Resuscitation and the Resuscitation Council of Southern Africa (RCSA) published the 2005 International Consensus on Cardiopulmonary Resuscitation and Emergency Cardiovascular Care Science with Treatment Recommendations.^3^,^4^

Based on this extensive literature review, what has been shown to improve survival?

## Rapid defibrillation

For every minute that the time to defibrillation is reduced, a 10 to 23% survival benefit can be achieved.^5^ Survival rates of more than 80% have be achieved when defibrillation occurs within a few minutes, as has been shown in cardiac rehabilitation centres.^6^ Implantable cardioverter/defibrillators are able to abort sudden death effectively in almost 100% of patients in whom they are implanted.^7^ Modern manual biphasic defibrillators have a first-shock efficacy of more than 90%.^8^ Immediate resumption of cardiopulmonary resuscitation (CPR) for two minutes after shock delivery, starting with compressions, is more likely to be beneficial than delivering a series of repeated (stacked) shocks before resuming CPR.

## Minimising interruptions in chest compressions

An analysis of 868 defibrillation waveform recordings showed a 90% reduction in successful defibrillation when chest compression ‘hands-off’ time was more than 20 seconds.^9^ Chest compressions should ideally, therefore, be performed before, during (particularly if a mechanical compression device is available) and immediately after shock delivery. Following successful termination of ventricular fibrillation (VF) or pulseless ventricular tachycardia (VT) by defibrillation, patients will still have a non-perfusing rhythm [pulseless electrical activity (PEA) or asystole].^10^ Therefore, it is important to continue chest compressions for at least two minutes following shock delivery before analysing the rhythm or checking for return of circulation.

## Press much more; blow much less

A recent observational study of 4 068 bystander-witnessed cardiac arrests in the Kanto region of Japan showed that performing ‘chest compressions only’ was more effective than conventional CPR using a 15:2 compression:ventilation ratio.^11^ A physiological and mathematical analysis of optimum compression:ventilation ratios suggested that converting from a 15:2 to a 30:2 ratio could result in a seven to 33% improvement in oxygen delivery.^12^

The quality of CPR currently being performed, both pre-hospital and in-hospital, is sobering. A study recording the quality of CPR performed by paramedics showed that chest compressions were not given 48% of the time, and only 28% of compressions that were done were of the correct depth.^13^ In-hospital resuscitation standards were no better, with chest compressions being too shallow in 38% of cases, and ventilation rates of more than 20 per minute in 61% of cases.^14^ Excessive ventilation rates (more than 12 per minute) are associated with poor survival.^15^

## Mechanical chest compression devices

As a result of several studies demonstrating that CPR is generally performed very poorly, as well as the common tendency to interrupt compressions frequently and for prolonged periods, interest in the use of mechanical chest-compression devices is steadily increasing. Two devices in particular, a battery-operated automated load-distributing band chest-compression device (AutoPulse; Zoll) and a compressed air-driven automated active compression:decompression piston system (LUCAS; Physio-Control) are both being investigated with intense interest.

## The role of vasopressors and anti-arrhythmics

There is no evidence that routine administration of any drug during cardiac arrest has improved long-term survival. In one study, adrenaline (and intubation) was associated with lower survival when 10 966 patients were evaluated one month post cardiac arrest.^16^ Furthermore, in a recent study involving 1 296 out-of-hospital cardiac arrest patients in Singapore, there was no survival-to-discharge benefit in patients receiving adrenaline compared with those that did not receive adrenaline.^17^ However, amiodarone has been demonstrated to improve short-term outcome in VF and pulseless VT.^18^,^19^

Of greater importance than drugs in cardiovascular resuscitation is an aggressive search for contributory factors and reversible causes. These can conveniently be memorised as six ‘Hs’ and six ‘Ts’, namely hypoxia, hypovolaemia, hydrogen ion excess (acidosis), hyper-/hypokalaemia, hypoglycaemia, hypothermia, and tension pneumothorax, tamponade, thrombosis (cardiac), thrombosis (pulmonary), toxins and trauma.^20^

## Induced (therapeutic) hypothermia

Probably the most promising of all interventions is the significantly improved neurological outcome among comatose survivors of pre-hospital VF cardiac arrest when cooled to between 32 and 34°C for 12 to 24 hours.^21^,^22^ Numerous devices and techniques are now commercially available to support this successful intervention. Induced hypothermia is rapidly becoming regarded as the ‘standard of care’ post-arrest in view of the ever-growing number of successful outcomes.

## Cardiac arrest algorithm

**Fig. 1.**
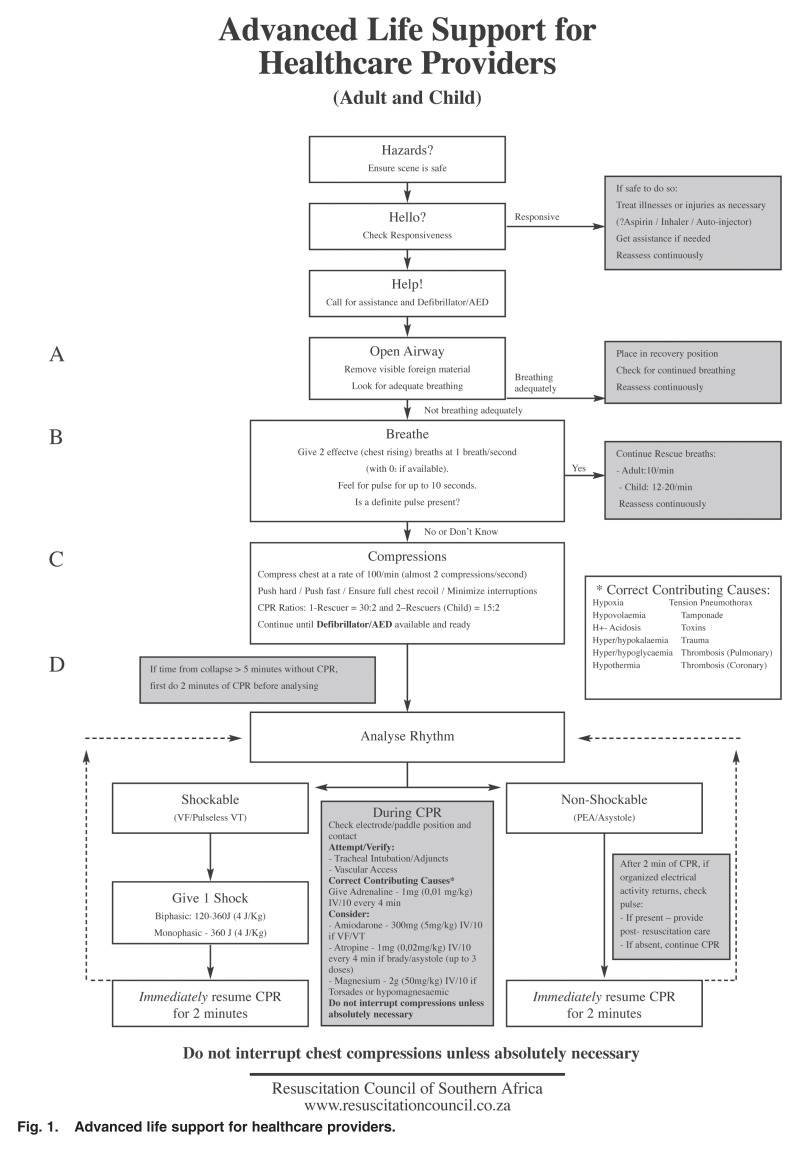
Advanced life support for healthcare providers.

A ‘universal cardiac arrest algorithm’, applicable to adult, child and infant resuscitation (excluding newborns), was designed by ILCOR to reflect the major changes in resuscitation recommendations, placed in chronological sequence.^3^,^4^ Resuscitation councils worldwide have subsequently based their current guidelines on these recommendations, with minor regional variations where appropriate. The RCSA, being a founder member of ILCOR in 1992, has been intimately involved in the international collaboration leading to the publication of 18 scientific advisory statements as well as the 2005 International Consensus on Cardiopulmonary Resuscitation and Emergency Cardiovascular Care Science with Treatment Recommendations.^23^ In taking into consideration the factors that have been shown to improve cardiac arrest survival, the RCSA, together with the Emergency Medicine Society of South Africa, posted onto its website (www.resuscitationcouncil.co.za) an algorithm on advanced life support for healthcare providers (Fig. 1).

The algorithm, which is applicable to both adult and paediatric victims, incorporates the principle of early defibrillation by calling for a manual or automated external defibrillator (AED) the moment the victim is found to be unresponsive. After administering two initial ventilations, if a definite pulse is not felt within 10 seconds, chest compressions are provided at a rate of 100 per minute (almost two compressions per second). High-quality compressions are stressed, with an emphasis on pushing hard, pushing fast, ensuring full chest recoil and minimising interruptions. A compression:ventilation ratio of 30:2 is recommended, with the only exception being a child victim, whereby two rescuers may perform a ratio of 15:2 to allow for slightly more ventilations. CPR must be continued until a defibrillator/AED becomes available and is ready for use. If ventilations cannot be provided for any reason, just perform continuous compressions until a defibrillator is attached.

Rhythm analysis in cardiac arrest is exceptionally simple. The rhythm is determined to be either ‘shockable’ (ventricular fibrillation or pulseless ventricular tachycardia) or ‘non-shockable’ (pulseless electrical activity or asystole). A single-shock strategy, followed immediately by CPR for two minutes is recommended for shockable rhythms. If the arrest was unwitnessed or a period of more than five minutes occurred without CPR being performed, it is suggested that two minutes of CPR be done prior to analysing the rhythm, in an attempt to supply blood and oxygen to the heart prior to attempting defibrillation.

If organised electrical activity is seen on the monitor after two minutes of CPR, one can check for a return of pulse. Pulse checks should not be done with ventricular fibrillation or asystole, as this will prolong the interruptions in compressions. During CPR it is important to check the electrode contacts and cable connections to ensure that artefact is not present on the monitor screen. Intravascular access and tracheal intubation or the insertion of airway adjuncts should be performed without interrupting CPR. Once an invasive airway device, such as an endotracheal tube or laryngeal mask airway has been inserted, ventilations can be provided once every five to six seconds without interrupting chest compressions.

Paramount to a successful resuscitation outcome is an aggressive search for, and the rapid correction of contributory causes of the cardiac arrest. The six ‘Hs’ and six ‘Ts’ memory aid referred to above can be used to assist in this regard. Emphasis is again laid on the need to minimise interruptions in chest compressions during this process.

Although now of debatable value, 1 mg adrenaline (0.01 mg/kg in children) can be given intravenously (IV) or intra-osseously (IO) every three to five minutes during CPR. If a shockable rhythm is present, 300 mg amiodarone IV/IO (5 mg/kg for children) should be considered. A second dose of 150 mg can be considered in adults. If torsades de pointes or hypomagnesaemia is suspected, 2 g magnesium IV/IO (50 mg/kg in children) should be considered. Suspect hypomagnesaemia in patients who are alcoholic, malnourished or elderly. Although 1–3 mg atropine may be given for asystole or slow PEA, its value in cardiac arrest is also debatable.

## Future directions?

A novel approach to resuscitation has very recently been reported in Arizona, USA.^24^ Out-of-hospital cardiac arrest patients received an initial series of 200 uninterrupted chest compressions, then a single defibrillation shock (if indicated), followed by another 200 immediate post-shock compressions before rhythm re-analysis. The cycle was repeated three times, that is, for the first six minutes. Passive ventilation was initially advocated by means of a non-rebreather facemask with high-flow 100% oxygen and insertion of an oropharyngeal airway. Intubation was delayed until after three cycles (six minutes) of compressions and rhythm analysis. Of 2 460 patients, overall survival to hospital discharge increased from 3.8 to 9.1%. Survival of patients with VF witnessed arrest increased from 4.7 to 17.6%. Another study done in a rural setting showed a tripling of neurologically intact survival, from 15 to 48% for patients receiving continuous chest-compression CPR following a witnessed out-of-hospital VF cardiac arrest.^25^

## The bottom line…

Only three interventions have been shown to unequivocally save lives following cardiac arrest:

● high-quality CPR, with emphasis on minimal interruptions in chest compressions
● early detection and defibrillation of a shockable rhythm
● rapid identification and correction of the contributory causes of the cardiac arrest.

After 45 years of research, the resuscitation paradigm has been shifted and there is thankfully now light at the end of the tunnel.
